# Absence of evidence of Xenotropic Murine Leukemia Virus-related virus infection in persons with Chronic Fatigue Syndrome and healthy controls in the United States

**DOI:** 10.1186/1742-4690-7-57

**Published:** 2010-07-01

**Authors:** William M Switzer, Hongwei Jia, Oliver Hohn, HaoQiang Zheng, Shaohua Tang, Anupama Shankar, Norbert Bannert, Graham Simmons, R Michael Hendry, Virginia R Falkenberg, William C Reeves, Walid Heneine

**Affiliations:** 1 Laboratory Branch, Division of HIV/AIDS Prevention, National Center for HIV/AIDS, Viral Hepatitis, STD, and TB Prevention, Centers for Disease Control and Prevention, Atlanta, GA 30333, USA; 2 Robert Koch-Institute, Centre for Biological Safety 4, Nordufer 20, 13353 Berlin, Germany; 3 Blood Systems Research Institute and Department of Laboratory Medicine, UCSF, 270 Masonic Ave., San Francisco, CA 94118, USA; 4 Chronic Viral Diseases Branch, Division of Viral and Rickettsial Diseases, National Center for Zoonotic, Vector-Borne and Enteric Diseases, Centers for Disease Control and Prevention, Atlanta, GA 30333, USA

## Abstract

**Background:**

XMRV, a xenotropic murine leukemia virus (MuLV)-related virus, was recently identified by PCR testing in 67% of persons with chronic fatigue syndrome (CFS) and in 3.7% of healthy persons from the United States. To investigate the association of XMRV with CFS we tested blood specimens from 51 persons with CFS and 56 healthy persons from the US for evidence of XMRV infection by using serologic and molecular assays. Blinded PCR and serologic testing were performed at the US Centers for Disease Control and Prevention (CDC) and at two additional laboratories.

**Results:**

Archived blood specimens were tested from persons with CFS defined by the 1994 international research case definition and matched healthy controls from Wichita, Kansas and metropolitan, urban, and rural Georgia populations. Serologic testing at CDC utilized a Western blot (WB) assay that showed excellent sensitivity to MuLV and XMRV polyclonal or monoclonal antibodies, and no reactivity on sera from 121 US blood donors or 26 HTLV-and HIV-infected sera. Plasma from 51 CFS cases and plasma from 53 controls were all WB negative. Additional blinded screening of the 51 cases and 53 controls at the Robert Koch Institute using an ELISA employing recombinant Gag and Env XMRV proteins identified weak seroreactivity in one CFS case and a healthy control, which was not confirmed by immunofluorescence. PCR testing at CDC employed a *gag *and a *pol *nested PCR assay with a detection threshold of 10 copies in 1 ug of human DNA. DNA specimens from 50 CFS patients and 56 controls and 41 US blood donors were all PCR-negative. Blinded testing by a second nested gag PCR assay at the Blood Systems Research Institute was also negative for DNA specimens from the 50 CFS cases and 56 controls.

**Conclusions:**

We did not find any evidence of infection with XMRV in our U.S. study population of CFS patients or healthy controls by using multiple molecular and serologic assays. These data do not support an association of XMRV with CFS.

## Background

Chronic fatigue syndrome (CFS) is a complex illness that affects between 0.5 and 2 percent of adults in the U.S. [1,2]. CFS is characterized by a severe debilitating fatigue lasting at least six consecutive months that is not alleviated with rest. Individuals with CFS also report various cognitive, sleep and musculoskeletal pain disturbances, and symptoms similar to those of infectious diseases [3]. At least a quarter of those suffering from CFS are unemployed or receiving disability because of the illness; the average affected family forgoes $20,000 annually in lost earnings and wages; and, the annual value of lost productivity in the United States is at least $9 billion [2,4-6]. Diagnostic, treatment, and prevention strategies have proven difficult to devise because the etiology, pathophysiology and risk factors for CFS remain unclear [3,7].

Because the symptoms characterizing CFS resemble those of infectious diseases, many studies have investigated a viral etiology in CFS. However, involvement of several viruses including human herpes virus-6 (HHV-6), Epstein-Barr virus (EBV), various enteroviruses, and the human T-lymphotropic virus type 2 (HTLV-2) has not been conclusively proven [3,7-10]. In October 2009, Lombardi *et al. *reported finding a gammaretrovirus called xenotropic murine leukemia virus-related virus (XMRV) in peripheral blood mononuclear cell (PBMC) DNA from about 67% (68/101) of CFS patients compared to only 3.6% (5/218) of healthy persons using PCR testing [11]. Virus isolation and antibody detection were also reported in some CFS patients [11].

XMRV is phylogenetically related to the xenotropic murine leukemia viruses (MuLV) sharing about 94% nucleotide identity across the viral genome [12]. XMRV was initially identified in prostate tissues from about 10% of prostate cancer patients using microarray and PCR analysis [12]. XMRV prevalence in this study was higher in patients with an inherited mutation in the RNase L gene [12]. More recent studies examining XMRV prevalence in prostate tissues of patients with prostate cancer from the US and Europe have reported both negative and positive findings [13-15], highlighting the need for more studies to assess the role of XMRV in prostate cancer.

Confirmation of an association and etiologic role of XMRV in CFS is important because it could provide a useful diagnostic test and might lead to new treatment interventions. However, two recent studies of CFS patients from the United Kingdom using PCR testing alone or together with serologic testing reported negative XMRV results in 186 and 170 CFS patients, respectively [16,17]. XMRV was also not found by PCR testing of 32 CFS patients and 43 matched controls from the Netherlands [18]. Additional studies of different patient cohorts, including those from the US, are critical to better evaluate both a possible association of XMRV with CFS and a potential geographic link.

We describe here results from the first US study following the initial report by Lombardi *et al*. [11]. Testing of 51 specimens from CFS patients and 56 matched and healthy controls from the US was performed independently in three laboratories for XMRV DNA by using several PCR tests and for anti-XMRV antibodies using different serological assays.

## Results

### Absence of XMRV antibodies in persons with CFS and healthy controls

**Figure 1:**
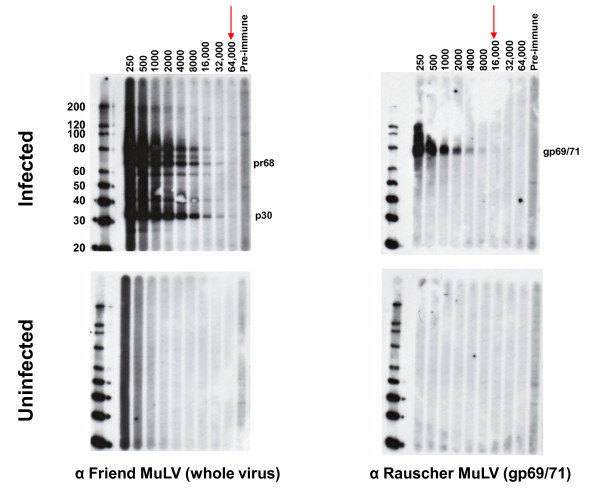
**Titration of polyclonal MuLV goat antisera in Western blot (WB) assay**. Antibody titers of positive control anti-sera and reactivity of pre-immune sera to polytropic MuLV-infected (upper panel) and uninfected (lower panel) HeLa cell crude cell lysates in WB testing. Specific antisera tested are located at the bottom of each WB. Arrows indicate observed titers for each antiserum. Fr, Friend; Ra, Rauscher. Locations of reactivity to specific viral proteins are indicated. Env (gp69/71), envelope; TM (p15E), transmembrane; MA (p15), matrix; Gag (pr68/80); CA (p30), capsid. Molecular weight markers (kD) are provided on the left of the WBs in the upper panels. Sizes of expected viral proteins are provided in each WB in the upper panels.

**Figure 2:**
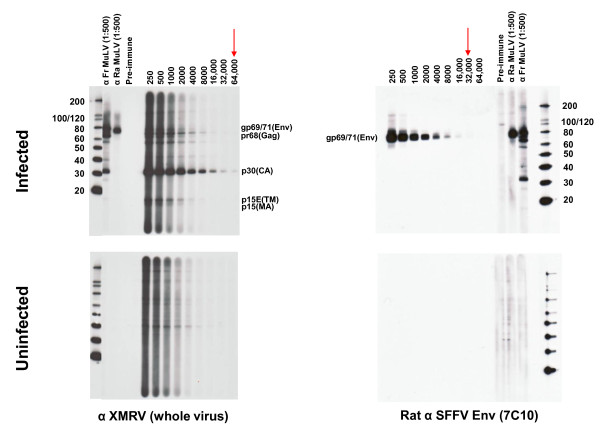
**Titration of polyclonal XMRV rabbit and monoclonal spleen focus forming virus (SFFV) envelope rat antisera in Western blot (WB) assay**. Antibody titers of positive control anti-sera and reactivity of pre-immune sera to polytropic MuLV-infected (upper panel) and uninfected (lower panel) HeLa cell crude cell lysates in WB testing. Specific antisera tested are located at the bottom of each WB. Arrows indicate observed titers for each antiserum. Fr, Friend; Ra, Rauscher. Locations of reactivity to specific viral proteins are indicated. Env (gp69/71), envelope; TM (p15E), transmembrane; MA (p15), matrix; Gag (pr68/80); CA (p30), capsid. Molecular weight markers (kD) are provided on the left of the WBs in the upper panels. Sizes of expected viral proteins are provided in each WB in the upper panels.

Serologic testing at CDC was performed with a newly developed WB assay using a strategy employed successfully for assessing human infection with other zoonotic retroviruses [19,20]. The WB test used lysate from polytropic MuLV (PMLV)-infected HeLa cells as antigen. PMLV and XMRV are highly related. They share between 87 and 93% nucleotide identity across the genome with XMRV and also have 88 - 97% and 88 - 91% amino acid identity to XMRV Gag and Env proteins, respectively. Partial Gag (123 aa) and Env (55 aa) sequences from our polytropic HeLa isolate share 96% and 90% identity to XMRV, respectively. Thus, excellent antigenic cross-reactivity between XMRV and our polytropic HeLa isolate is expected. Specimens were tested for reactivity in parallel against control antigens from uninfected HeLa cell lysates. Positive seroreactivity was defined as detection of bands in the infected lysates corresponding to known viral antigens and a lack of similar reactivity in uninfected lysates to exclude nonspecific reactivity. Four available antisera demonstrated good antigenic reactivity to Gag and/or Env proteins (Figures 1 and 2): Goat anti-MuLV polyclonal antisera to whole virus and to p69/71 Env proteins, rabbit anti-XMRV polyclonal antiserum to whole virus, and rat monoclonal antibody to the Env of spleen focus forming virus (SFFV), a polytropic MuLV, that reacts with gp69/71 Env of polytropic and xenotropic MuLV [21]. The anti-XMRV antiserum was used previously to detect XMRV in prostate cancer tissues by immunohistochemistry [13]. The anti-SFFV antibody was used by Lombardi *et al*. in a flow-based antibody competition assay to detect antibodies to XMRV Env in CFS patients [11]. All positive control antisera were reactive at high titers to various Gag and/or Env proteins (Figures 1 and 2). The anti-MuLV whole virus antiserum and the anti-XMRV polyclonal antiserum both reacted to the p68/p80 Gag precursor and p30 Gag proteins at titers of 1:32,000 and 1:64,000 respectively (Figures 1 and 2). The polyclonal anti-gp69/71 Env antiserum and the anti-SFFV monoclonal antibody reacted with the Env gp69/71 doublet proteins (Figures. 1 and 2) at a titer of 1:8,000 and 1:32,000, respectively (Figures. 1 and 2). The same pattern of reactivity was seen using both the anti-MuLV whole virus and anti-XMRV antisera though a higher level of nonspecific reactivity was observed to the HeLa lysates with the XMRV antisera (Figures 1 and 2). No specific reactivity was observed for the pre-immune goat sera and to uninfected HeLa lysates (Figures 1 and 2). 1:500 dilutions of the whole virus and gp69/71 antisera and a 1:50 dilution of pre-immune goat sera were then used as positive and negative controls for testing patient samples in the WB assay, respectively.

**Figure 3:**
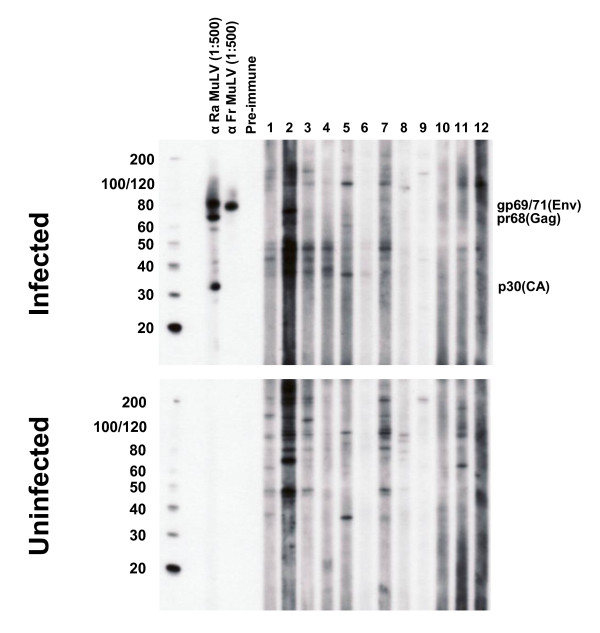
**Absence of XMRV antibodies in CFS patients by Western blot (WB) analysis**. Representative WB results for CFS cases from Wichita and Georgia identified after unblinding. Determination of MuLV specific reactivity is determined by comparison of observed seroreactivity to polytropic MuLV-infected HeLa antigens and uninfected HeLa antigens in upper and lower panels, respectively. Lanes 1 - 4 and 5 - 8 are plasma from CFS cases from the population based studies in Georgia and Wichita, respectively; lanes 9 - 12 are physician-referred CFS cases from the Georgia Registry study. MuLV positive and negative goat serum controls are labelled.

Plasma samples from 51 CFS cases and 53 healthy controls were diluted 1:50 and examined for seroreactivity to bands corresponding to Gag (p30 or p68/80) and/or Env (gp69/71 or p15E) proteins present in only the infected lysate and not the uninfected lysate. We also tested sera from 26 retrovirus-positive specimens (13 HTLV-1/2, seven HIV-1, and six dual HIV-1/HIV-2 seropositive patients) and observed no reactivity to XMRV proteins (data not shown) confirming a lack of cross-seroreactivity. In addition, we tested archived sera from 121 anonymous US blood donors; all were negative (data not shown). Plasma samples from the 51 CFS patients and 53 healthy controls all tested negative for XMRV antibodies in this assay. Plasma samples were not available from three healthy controls. Typical WB results of CFS persons are shown in Figure 3. Every plasma specimen demonstrated some level of background reactivity, but without evidence of specific reactivity to Gag and/or Env proteins (Figure 3). For example, plasma from a CFS person showed reactivity to two proteins about 65 and 69 kD in size in the infected cell lysate but reacted non-specifically to proteins of the same size in the uninfected antigen and was thus considered seronegative (lane 2 of Figure 3). There were no clear differences in nonspecific WB seroreactivity observed in healthy persons compared to persons with CFS (data not shown).

**Figure 4:**
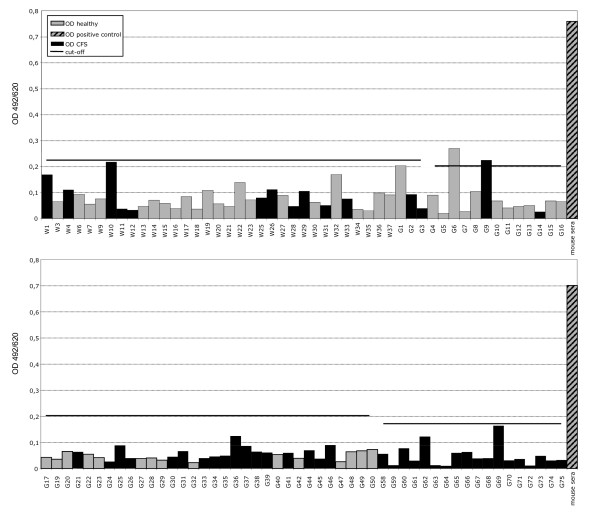
**Absence of XMRV antibodies in CFS patients and healthy persons by ELISA using recombinant XMRV proteins**. Representative XMRV Envelope (Env) ELISA results for 50 CFS cases and 49 healthy persons identified after unblinding. Specimens coded with W and G1-G50 are from the population-based study in Wichita and Georgia, respectively; specimens G59 - G75 are from physician-referred CFS cases from the Georgia Registry study. Specimens from a healthy control and a person with CFS, coded as G6 and G9 respectively, were weakly seroreactive in this test but were not confirmed by either Western blot or immunofluorscence testing. Human sera were diluted 1:200. The human negative control serum was obtained from a healthy volunteer previously determined to be seronegative. The polyclonal mouse Env antiserum was diluted 1:100. Assay cut-off was determined by the mean of the test samples plus three standard deviations.

Blinded serologic testing of these same CFS and control specimens was also performed at the Robert Koch Institute (RKI) in Germany using ELISAs containing recombinant XMRV Gag and Env proteins [14]. Plasma from 51 CFS cases and 53 healthy controls were not reactive in the recombinant XMRV Gag ELISA using either the N- or the C-terminus of the protein [14]. Two specimens, one each from a CFS patient (G9) and healthy control (G6), were weakly reactive in the recombinant XMRV Env ELISA with optical densities (OD) slightly above the assay cutoff of 0.2 OD units (Figure 4) [14]. However, both specimens were negative by IFA testing using 293T cells expressing either XMRV Gag or Env proteins and were thus considered negative. Two blinded positive control specimens each consisting of goat polyclonal MuLV whole virus antisera diluted 1:100 in pre-immune goat sera both tested positive in the recombinant Gag ELISAs but were negative in the Env ELISA. These results are consistent with the seroreactivity of these polyclonal antisera to only Gag proteins in the WB assay. Five undiluted pre-immune goat sera all tested negative in both the Gag and Env ELISAs. These "external" positive and negative controls were included as a separate set of specimens and were all correctly detected in a blinded fashion. Testing of the blinded human and goat control specimens was performed separately since different secondary antibody conjugates are used for these different specimens. Internal positive and negative controls were also included in each run and performed as expected. Like the WB testing, the goat anti-MuLV whole virus and anti-MuLV p70 polyclonal antisera gave titers of 1:64,000 and 1:6,400 in the Gag and Env ELISAs, respectively.

### Absence of XMRV sequences in PBMC DNA from persons with CFS and healthy controls

We used two PCR assays at CDC to detect XMRV DNA. The first assay was a nested *gag *PCR test used previously to identify XMRV sequences in prostate cancer patients and CFS patients [11,12]. The second nested PCR assay was designed on highly conserved polymerase (*pol*) sequences within xenotropic and other MuLV strains. Serial, ten-fold dilutions of full-length XMRV(VP62) plasmid (kindly provided by Robert Silverman) in a background of human DNA (PBMC or whole blood) showed that the nested *gag *and *pol *PCR tests each detected 10 XMRV copies in different experiments on subsequent days (34/34 (100%) and 32/34 (94.1%), respectively). These results show that both PCR assays have an excellent sensitivity for detecting XMRV in one ug of DNA specimen. PBMC DNA from 41 anonymous US blood donors was also tested and found to be negative in both PCR assays. These 41 blood donors are distinct from the US blood donors whose plasmas were tested in the WB test.

**Figure 5:**
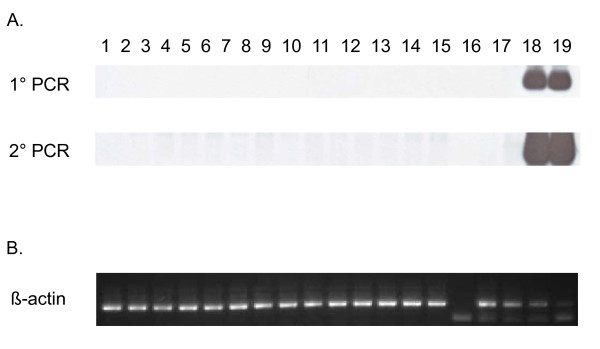
**Absence of XMRV polymerase (*pol*) sequences in CFS patients**. A. Representative nested *pol *PCR results using PBMC DNA specimens from persons with CFS identified after unblinding. Lanes 1 - 5, 6 - 10, and 11 - 14 are results for persons with CFS from Wichita, Georgia, and the Georgia registry studies, respectively; lanes 15 and 16, water only controls; lane 17, negative human PBMC DNA control; lanes 18 and 19, assay sensitivity controls consisting of 10^1 ^and 10^3 ^copies of XMRV VP62 plasmid DNA diluted in a background of 1 ug of human PBMC DNA, respectively. B. Semi-quantitative β-actin PCR results for PBMC DNA specimens above in lanes 1 - 14; lane 15, water control; lanes 16 - 19, 10-fold dilutions of blood donor PBMC DNA starting at 0.1 ug as a positive assay control.

PCR testing of β-actin sequences was positive for all clinical specimens confirming the integrity of the DNA and an absence of PCR inhibitors. Representative β-actin PCR results are shown in Figure 5. Subsequent XMRV testing showed that XMRV *gag *and *pol *sequences were not detected in 1 ug of PBMC (n = 31) or whole blood (n = 19) DNA from the CFS patients or in 1 ug PBMC DNA from the 56 healthy controls. A representative Southern blot of the XMRV *pol *PCR testing of persons with CFS is shown in Figure 5. Matching DNA was not available from one CFS case.

**Figure 6:**
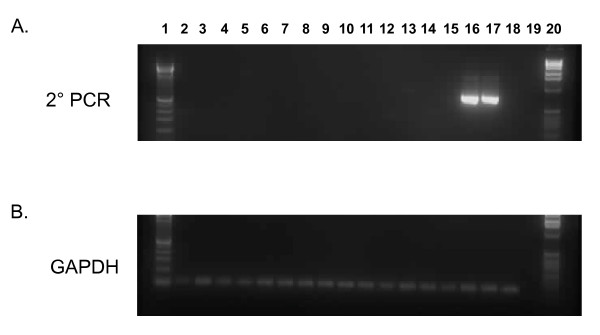
**Absence of XMRV *gag *sequences in CFS patients**. A. Representative nested *gag *PCR results from patients from the Georgia Registry identified after unblinding. Lanes 1 and 20, 100-bp ladder; lanes 2 - 15 are results from CFS patients; lanes 16 - 18 assay sensitivity controls consisting of 10, 3 and 1 copies of XMRV VP62 plasmid DNA diluted in a background of 250 ng of human PBMC DNA; lane 19, water control. B. GAPDH PCR results for same PBMC DNA specimens above.

Blinded PCR testing performed at an independent institution (Blood Systems Research Institute (BSRI), CA) using a second nested PCR assay for XMRV *gag *DNA sequences, with a sensitivity of 3 copies per reaction, was also negative using 100 ng DNA specimens from all 50 CFS cases and 56 healthy controls (data not shown). 250 ng of DNA from the Georgia Registry patients also tested negative using this nested *gag *PCR test (Figure 6). Four blinded, "external" control specimens, included with the panel of human specimens and spiked with 4, 40, 400, and 4000 XMRV plasmid copies in 100 ng of human DNA, were all detected by this testing (data not shown).

## Discussion

We found no evidence of infection with XMRV among persons with CFS or matched healthy controls from the US by testing with multiple serologic and PCR assays performed independently in three laboratories blinded to the clinical status of the study participants. Our results contrast with the high rate of XMRV detection reported by Lombardi *et al. *among both CFS patients and controls, but are in agreement with recent data reported in two large studies in the UK and a smaller study in the Netherlands that could not detect XMRV sequences in CFS patients and one UK study that also failed to detect specific XMRV neutralizing antibody responses in CFS [11,16-18]. Combined, these negative data do not support XMRV as the etiologic agent of the majority of CFS cases.

Several possibilities could explain these discordant results, including technical differences in assays used for the testing in each study. However, the inability of four independent laboratories to replicate the high XMRV prevalence in CFS cases reported by Lombardi *et al. *cannot be explained by minor differences in assays used in each study. In addition, testing at CDC utilized the nested XMRV *gag *PCR assay used by Lombardi *et al. *and Urisman *et al. *to identify XMRV infection in CFS and prostate cancer patients, respectively [11,12]. Further, to improve assay sensitivity, we used 1 ug of input DNA which is 4-5 times higher than that used by others [11-13,16,17], all while maintaining an assay sensitivity of 10 copies. To ensure that our testing would not miss genetically diverse XMRV or MuLV strains, we also used a sensitive nested PCR assay with conserved *pol *gene primers and found that this testing was also negative confirming the absence of XMRV/MuLV sequences. While PBMC DNA was used in the majority of specimens, 1 ug whole blood DNA was also used in testing 19 CFS cases. This input DNA represents about 350 ng of PBMC DNA which is similar to the amount used by others [11-13,15,16], thus not affecting the sensitivity of our results. The negative PCR findings were confirmed by an independent laboratory with a second nested *gag *PCR assay which provided additional evidence for the absence of XMRV sequences among CFS cases and controls. The primary PCR amplification used in this second test is also that used by Lombardi *et al. *which when combined with a nested PCR step has a 3-copy detection threshold.

Antibody responses particularly to Gag and Env proteins are hallmarks of immune responses to retroviral infections including experimental XMRV infection of macaques [22]. We used a new WB assay to test for anti-XMRV antibodies and showed by using both monoclonal antibodies and polyclonal antisera that this assay detected specifically, and with high titers, reactivity to both XMRV and MuLV Gag and Env proteins. We were unable to detect antibodies to XMRV Gag and Env in any of the CFS and controls specimens by using this WB assay. Likewise, negative results were obtained in a second, independent laboratory by using XMRV-specific ELISA-based and IFA assays. Thus, the observed negative serologic results for all CFS patients reflect an absence of antibody responses and active XMRV infection. Although limited, the negative WB serology observed in 56 healthy controls and 121 blood donors also suggests that the XMRV seroprevalence in this population is not high. Screening of larger numbers of US blood donors using a high throughput ELISA followed by confirmation in a WB test also showed uncommon seropositivity (~0.1%) [22]. More studies, however, are needed to determine the prevalence of XMRV in healthy populations.

One current limitation of our study, and of others performing serologic and PCR testing for XMRV, is the absence of bona fide positive and negative control specimens from infected and uninfected humans to determine the analytical sensitivity and specificity of the detection assays. Until panels of well-characterized clinical specimens become available, assay validation will be limited to reagents generated experimentally, such as polyclonal and monoclonal antibodies, XMRV plasmids, and XMRV-infected cells.

The selection criteria with which persons with CFS were included in these various studies may also help to explain the incongruent XMRV findings. The study by Lombardi *et al. *used samples from the *Whittemore Peterson Institute National Tissue Repository *reported to contain specimens from well-characterized cohorts of CFS [11]. Yet, the paper provides no information regarding the repository or concerning the nature of these cohorts other than that they were collected from private medical practices in several regions of the U.S. where clusters of CFS have been documented [11]. An absence of details of the CFS cases and controls in this report makes it difficult to replicate and interpret their findings. In contrast, patients in the UK and Netherland studies were typical of CFS patients seen in specialist clinical services in those countries and resemble persons seen in other specialist CFS services in the US and Australia [16-18]. Almost half of the UK CFS patients described onset of their illness as related to an acute viral disease [16,17]. Thus, they would appear quite comparable to those in the study by Lombardi *et al. *Similarly, our study also failed to detect XMRV infection in 18 CFS patients referred to a fatigue registry by health care providers in Georgia and included three persons who reported sudden onset to their illness. Our study is the first to evaluate XMRV infection in persons with CFS and healthy controls from the general populations of Wichita and Georgia. These CFS cases are different from CFS patients seen in general practice and referral clinics; of the participants from the population-based study in Georgia, only half had consulted a physician because of their fatigue, about 16% had been diagnosed with CFS, and 75% described an insidious onset to their illness that had no obvious relation to an acute infectious disease. Nonetheless, results from our general population cohort extend the examination of XMRV in CFS to persons whose illness developed gradually, for the most part, rather than acutely. Our negative findings, in conjunction with those in Europe [16-18], indicate no discernable association of XMRV with a wide spectrum of CFS cases. The negative results for CFS patients and controls from the US in the current study also do not support a continental clustering of XMRV infection suggested by the absence of infection in the UK and Netherlands [16-18]. However, our findings may not be generalizable beyond our study populations because XMRV infection rates may vary in different regions or locales.

CFS is a diagnosis of exclusion based on self-reported symptoms and requires careful medical and psychiatric evaluations to rule out conditions with similar clinical presentation. Our study and the negative reports from the UK and the Netherlands evaluated patients for exclusionary conditions and defined CFS according to criteria of the 1994 International CFS Research Case Definition [23] or the earlier Oxford case definition [24]. The Lombardi *et al*. study specifies that samples were selected from patients fulfilling the 1994 international CFS case definition [23] and the 2003 Canadian Consensus Criteria for CFS/ME [25]. Lombardi *et al. *did not specify if patients were evaluated for exclusionary conditions, or if the study subjects met both definitions, or which patients met either CFS definition. The 1994 International CFS case definition and the Canadian Consensus Criteria are different and do not necessarily identify similar groups of ill persons. Most notably, the Canadian Criteria include multiple abnormal physical findings such as spatial instability, ataxia, muscle weakness and fasciculation, restless leg syndrome, and tender lymphadenopathy. The physical findings in persons meeting the Canadian definition may signal the presence of a neurologic condition considered exclusionary for CFS and thus the XMRV positive persons in the Lombardi *et al. *study may represent a clinical subset of patients [11].

CFS is a complex disease with various clinical subtypes proposed which could also account for differences in the results obtained in each study [11,16-18]. While there is still no universal agreement on a precise clinical presentation encompassing CFS illness, defining patient characteristics in studies of CFS etiology or pathogenesis remains crucial for making comparisons across various research conclusions.

## Conclusions

In our study population of CFS and healthy persons from the US, we did not find any evidence of infection with XMRV using PCR and serologic methods performed independently in three laboratories blinded to the clinical status of the study participants. These results do not support an association of XMRV with CFS.

## Methods

### Study population and specimen preparation

The CDC Institutional Review Board reviewed and approved all study protocols. All participants were volunteers and provided informed consent. Laboratory testing of the samples was performed anonymously and blinded to clinical status.

Details of our two study populations have been described previously [2,26,27]. Briefly, between 2002 and 2003 we sampled adults 18 to 59 years old from Wichita, Kansas [26,27] and between 2008 and 2009 we sampled adults 18 to 59 years old from metropolitan, urban, and rural Georgia [2]. In both studies, we used random digit-dial screening interviews to classify household residents as either well or having symptoms of CFS. A follow-up detailed telephone interview was administered to all individuals with symptoms and to a probability sample of those without symptoms. Based on the detailed interview, those meeting criteria of the 1994 International CFS Research Case Definition [23] were classified as CFS-like and other respondents classified as either unwell (not CFS-like) or well. All CFS-like individuals were recruited and a random sample of those who were unwell but not CFS-like, and a set of matched (sex, age, race/ethnicity, geographic) well people were recruited for a 1-day clinical evaluation.

We also tested specimens from CFS cases identified in a CDC Health Care Provider-based Registry of Unexplained Fatiguing Illnesses and CFS (unpublished)*. *Between October 2008 and December 2009, healthcare providers practicing in Bibb County, GA referred adolescents and adults 12 - 59 years old who met criteria for unexplained fatiguing illness (fatigue for > 1 month), and having at least one other core CFS symptom during that period (unrefreshing sleep, problems with cognition or memory, joint or muscle pain in extremities), and did not have an exclusionary medical or psychiatric condition. All referred patients underwent a telephone screening interview to document fatigue lasting > 6 months, and the presence of at least one core symptom and no exclusionary conditions. Patients meeting these criteria underwent the same 1-day clinical evaluation as persons from our population-based studies, described in detail below.

### Clinical assessment

Clinical evaluations involved: 1. Administration of standardized questionnaires to measure the 3 domains of the 1994 CFS case definition [23]: the Multidimensional Fatigue Inventory (MFI) to measure 5 dimensions of fatigue [28] the Medical Outcomes Survey Short Form 36 (SF-36) to evaluate 8 dimensions of functional impairment [29]; and the CDC Symptom Inventory to evaluate occurrence/frequency/severity of the 8 CFS-accompanying symptoms [30]; 2. A standardized physical examination conducted by a specifically trained physician who also reviewed past medical history, review of systems, and current medications/supplements; 3. Collection of blood and urine for routine clinical analyses [23,31]; 4. A standardized psychiatric evaluation conducted by specifically trained psychiatric interviewers - Diagnostic Interview Schedule (DIS) in Wichita [32] and the Structured Clinical Interview for DSM-IV Disorders (SCID) in Georgia [33].

The physician's evaluation and routine clinical laboratory tests served to identify medical conditions considered exclusionary for CFS, specified in the 1994 case definition [23] as further clarified by the International CFS Study Group in 2003 [31]. The psychiatric interview served to identify current psychiatric disorders considered exclusionary for CFS, which included current melancholic depression, current or lifetime bipolar disorder or psychosis, substance abuse within 2 years and eating disorders within 5 years [23,31].

### Illness classification

Following clinical evaluation, participants who had no exclusionary medical or psychiatric conditions were diagnosed with CFS if they met criteria of the 1994 international case definition [23] as quantified by the CDC Symptom Inventory and ancillary criteria of the MFI and SF-36 [26,31]. We used the MFI to assess fatigue status [28]. For classification as CFS, those with a score ≥ well-population medians on the general fatigue or reduced activity scales of the MFI were considered to meet fatigue criteria of the 1994 international case definition. Functional impairment was assessed by the medical outcomes survey short form-36 (SF-36) [29]. For classification as CFS, those with a score ≤ 25th percentile of population norms in the physical function or role physical, or social function, or role emotional subscales of the SF-36 were considered to have substantial reduction in activities as specified in the 1994 definition. Those who met at least one but not all 1994 criteria were considered unwell not CFS. Those who met none of the criteria were considered well.

Specimens were available from 89 persons (33 CFS and 56 well controls) from the population-based case-control studies and 18 CFS persons from the Registry study described above. Subjects were included based on availability of specimens, and comprised 11 of 43 persons with CFS and 26 of 53 healthy controls from Wichita, KS and 22 of 32 persons with CFS and 30 of 51 healthy controls from Georgia. Persons with CFS and healthy controls had similar mean ages, similar predominance of females and white race, and had a similar mean body mass index (BMI) (Table 1). Subjects with CFS had been ill on average 13.9 years (median 11.15 yrs, range 3 - 40 yrs), were severely fatigued (MFI General Fatigue 16.5, range 10 - 20; MFI Reduced Activity 12.8, range 4 - 20) and severely impaired (SF-36 physical functioning 65.5, range 10-100); SF-36 bodily pain 48.8, range 12 - 84), and 3/33 (9%) reported sudden onset to their illness. Clinical and demographic characteristics of subjects with specimens available for this study did not differ from those persons who did not have ample specimen volumes and case-control matching was maintained.

**Table 1:** Distribution of demographic variables by CFS case-control status among persons from the combined Wichita and Georgia case-control population-based studies.

	CFS		Well		
**Demographic Factor**	**Wichita, KS** **(N = 11)**	**Atlanta, GA** **(N = 22)**	**Wichita, KS** **(N = 26)**	**Atlanta, GA** **(N = 30)**	**p-value**^**2,3,4**^

**Age**					
Mean ± SEM^1^	46.7 ± 3.32	47.7 ± 4.69	51.6 ± 5.1	46.1 ± 5.48	p = 0.51

**Sex [n (%)]**					
Female	8 (72.7)	20 (90.9)	21 (80.8)	25 (83.3)	p = 0.74
Male	3 (27.3)	2 (9.1)	5 (19.2)	5 (16.7)	

**Race [n (%)]**					
White	10 (90.9)	18 (81.8)	25 (96.2)	27 (90)	
Black	0 (0)	3 (13.6)	1 (3.8)	3 (10)	p = 0.69
Other	1 (9.1)	1 (4.6)	0	0 (0)	

**Body Mass Index**					
Mean ± SEM	27.6 ± 3.3	28.2 ± 4.7	29.2 ± 5.1	26.3 ± 5.5	p = 0.76

1. SEM, standard error of the mean

2. t-test was used to compute probabilities for comparisons of mean age and mean body mass index between study groups.

3. Chi square test was used to compute the probability for comparison of the distribution of sex between cases and controls.

4. Fisher's exact test was used to compute the probability for comparison of the distribution of race between the study groups, and was based on Blacks and Whites only.

18 of 38 persons enrolled in the Registry study had a diagnosis of CFS and were available for the current study. These provider-referred CFS patients had a mean age of 42.8 years (SEM = 2.85 years), and were predominantly white [17/18, (94.4%)] and female [16/18 (88.99%)]. They had suffered fatigue for an average of 9.4 years (range: 1 - 35 years) and 3/18 (16.7%) reported sudden onset to their illness.

### Specimen collection, processing, storage

Fresh whole blood was collected in either CPT Vacutainer tubes containing sodium citrate and a blood separation reagent (Becton Dickinson, NJ, USA) for the Georgia and Wichita studies or in PAXgene tubes for the Georgia CFS Registry study and transported to CDC. Blood was also collected in PAXgene tubes for two persons from the Georgia population-based study. PAXgene tubes were gently inverted 5 times, stored overnight at -20°C, and then transferred to -70°C until DNA isolation was performed. PBMCs and plasma were immediately isolated by centrifugation of the CPT tubes. PBMCs were stored in liquid nitrogen under conditions designed to maintain viability. Plasma was aliquoted and stored at -80°C within 4 hours of blood collection. For samples collected from persons living in Wichita, KS and from the Georgia CFS Registry study, whole blood was also collected in EDTA Vacutainer tubes. Plasma was recovered from the EDTA-treated blood by centrifugation at 15,000 × g for 20 minutes and aliquoted and frozen at -80°C until use. Plasma samples were aliquoted again when thawed for WB testing; the remaining aliquots were re-frozen at -80°C.

DNA was extracted from cryopreserved PBMCs or frozen whole blood with the Qiagen blood DNA minikit or Qiagen PAXgene Blood DNA kit (Qiagen, Valencia, CA), respectively, then aliquoted and stored frozen at -80°C. All PBMC samples had viabilities > 90% when they were thawed for DNA isolation. Nucleic acid concentrations were determined by spectrophotometry using the Nanodrop instrument (Thermo Scientific, Wilmington, DE). For the PCR testing at CDC, 1 ug of PBMC or whole blood DNA was used. Integrity of the DNA specimens was determined using β-actin PCR as previously described [34]. Matching plasma or DNA was not available from three healthy persons from Wichita, KS and one CFS case from Georgia, respectively. All specimen preparation, tissue culture, and PCR testing was done in physically isolated rooms to prevent contamination of specimens.

### Serologic Assays

HeLa cells were infected with supernatant from the murine macrophage cell line RAW264.7 (ATCC, Manassas, VI) known to express polytropic and ecotropic MuLV (PMLV and EMLV, respectively). To characterize the isolate that replicated in HeLa cells, a 166-bp RNA sequence containing the variable region C of the envelope (Env) surface protein was PCR-amplified from infected HeLa cell tissue culture supernatants. Phylogenetic analysis of the *env *sequence showed that the isolate was a PMLV by clustering tightly with other PMLV, and not EMLV (data not shown). XMRV and PMLV are highly related sharing between 87 - 94% nucleotide identity across their genomes and 88 - 97% and 88 - 91% amino acid identity to complete Gag and Env proteins, respectively. Indeed, partial Gag (123 aa) and Env (55 aa) sequences from our polytropic HeLa isolate share 96% and 90% identity to XMRV, respectively. Thus, the high amino acid relatedness supports the use of this isolate for WB serologic testing. Infected and uninfected HeLa crude cell lysates were prepared for WB testing as previously described [35]. Protein concentrations of the lysates were determined using the BioRad DC Protein Assay (Hercules, CA). Plasma or serum samples were diluted 1:50 and reacted separately to 150 ug of infected and uninfected cell lysates overnight at 4°C after protein separation through 4-12% polyacrylamide gels and transfer to nytran membranes, as previously described [35,36]. Seroreactivity in human specimens was detected using peroxidase-conjugated protein A/G (Pierce, Rockford, IL) and chemiluminescence (Amersham, Uppsala, Sweden) [35,36].

Since validated XMRV-positive human sera are not currently available, we used experimentally derived polyclonal antisera and monoclonal antibodies to assess antigenic reactivity of the WB assay. These reagents included goat polyclonal antisera to MuLV (whole virus and gp69/71Env, respectively) available at ATCC (VR-1537 and VR-1521, respectively), and a rabbit anti-XMRV polyclonal antiserum (kindly provided by Ila Singh) and a rat anti-SFFV (7C10) monoclonal antibody (kindly provided by Sandra Ruscetti) used previously to detect XMRV protein expression and antibodies in prostate cancer and CFS patients, respectively [11,13,21]. Peroxidase-conjugated protein A/G or anti-rat antibody (Sigma, St. Louis, MS) was used to detect bound goat, rabbit, and rat antibodies, respectively. Sensitivity of the assay was estimated using two fold serial dilutions of the MuLV, XMRV, and SFFV polyclonal and monoclonal antibodies. Cross-reactivity of the WB assay on HIV and HTLV positive plasma was evaluated on 13 HTLV-1/2 positive, 7 HIV-1-positive, and six HIV-1/HIV-2 dual positive plasma. In addition, sera from 121 HIV and HTLV seronegative anonymous US blood donors collected in 1998 were tested.

An aliquot of coded plasma from the CFS and healthy controls was tested at RKI by an ELISA using recombinant Gag and Env proteins used recently to investigate XMRV infection in German prostate cancer patients [14]. Briefly, recombinant proteins were coated overnight on microtiter plates at room temperature in equimolar amounts. The plates were blocked with 2% Marvel milk powder in phosphate buffered saline (PBS) for 2 h at 37°C, washed three times with PBS, 0.05% Tween 20. Patient plasma diluted 1:200 in PBS with 2% milk powder and 0.05% Tween20 were added into each well and incubated for 1 hour at 37°C. Each well was again washed three times and a 1:1000 dilution of a goat anti-human IgG-HRP conjugate (Sigma Aldrich, Munich, Germany) in PBS, 2% milk powder, 0.05% Tween 20 (Serva, Heidelberg, Germany) was added. Following incubation for 1 hour at 37°C, each well was again washed three times, and chromogen ortho-phenylendiamin (OPD) in 0.05 M phosphate-citrate buffer, pH 5.0 containing 4 μl of a 30% solution of the hydrogen peroxide substrate per 10 ml was added. After 5-10 minutes the color development was stopped by addition of sulphuric acid and the absorbance at 492 nm/620 nm was measured in a microplate reader. Positive controls included mouse anti-Gag and Env antisera and pre-immune sera diluted 1:50 in PBS with 2% milk powder and 0.05% Tween20. In addition, a separate set of goat sera was also tested in a blinded fashion and included external positive and negative controls consisting of dilutions of the MuLV whole virus, gp69/71 goat polyclonal antisera, or pre-immune goat sera, respectively. Detection of antibody reactivity in the goat sera was done by using rabbit anti-goat HRP conjugate (Dako, Hamburg, Germany).

Samples reactive by ELISA testing were then re-tested using an immunofluorescence assay (IFA) [14]. Briefly, plasma specimens were diluted 1:200 in blocking buffer and tested against 293T cells expressing codon optimized synthetic full-length genes of the XMRV *env *or *gag *under control of the CMV promoter and bound to glass slides, as described in detail previously [14]. Following incubation for 60 min at 37°C, the slides were washed extensively with PBS and secondary antibodies conjugated to fluorophores were added for 30 min. After thorough washing steps with PBS, the cells were mounted in Mowiol and viewed on a Zeiss (LSM510) confocal laser-scanning microscope.

### Detection of XMRV sequences

DNA specimens were screened by PCR at the CDC with an XMRV-specific *gag *and a polymerase (*pol*) assay that detects xenotropic and polytropic MuLV. The XMRV specific assay uses the primers GAG-O-F and GAG-O-R and GAG-I-F and GAG-I-R for the primary and nested PCRs, respectively, and conditions as previously described [11,12]. This is the same nested PCR test used by Urisman *et al. *and Lombardi *et al. *to detect 413-bp XMRV *gag *sequences in prostate cancer and CFS patients, respectively [11,12]. The primers and probes of the generic *pol *PCR assay were designed from an alignment of complete XMRV and prototypical xenotropic, polytropic, and ecotropic MuLV genomes available at GenBank (accession numbers: **xenotropic (XMLV): **XMRV VP35 = DQ241301, XMRV VP62 = DQ399707, XMRV VP42 = DQ241302, XMRV WPI-1106 = GQ497344, XMRV WPI-1178 = GC497343, MuLV DG-75 = AF221065; MuLV MTCR = NC_001702, mERV Chr 9 = AC121813, mERV Chr 4 = AL627077, mERV Chr 1 = AC083892; **polytropic (PMLV): **mERV Chr 7 = AC167978, mERV Chr 7 = AC127565, mERV Chr 12 = AC153658; **ecotropic (EMLV): **MuLV AKV = J01998, MuLV BM5eco = AY252102.1, Moloney MuLV = J02255, Rauscher MuLV = NC_001819, Friend MuLV = X02794). The external external XPOLOF (5' CCG TGC CCA ACC CTT ACA ACC TCT 3') and XPOLOR (5' CCG AGG TTC CCT AGG GTT TGT AAT 3') and internal primers XPOLIF (5' TCC ACC CCA CCA GTC AGC CTC TCT 3') and XPOLIR (5' AAG TGG CGG CCA GCA GTA AGT CAT 3') were used to generically detect 216-bp XMLV/XMRV *pol *sequences. All assays were optimized to achieve the highest sensitivity in detecting XMRV VP62 plasmid DNA in one ug of genomic DNA. One ug of human DNA was used as input for the PCR tests. PCR products were visualized by electrophoresis in an ethidium bromide-stained 1.8% agarose gel. To further increase the sensitivity and specificity of the PCR assays, amplified *gag *and *pol *sequences were confirmed by Southern blot analysis using the biotinylated oligoprobes XGAGP2 (5' ACC TTG CAG CAC TGG GGA GAT GTC 3'), and XPOLP (5' TTG ATG AGG CAC TGC ACA GAG ACC 3') and chemiluminescence detection. The detection limit of the assays was evaluated using 10-fold dilutions of XMRV VP62 plasmid diluted in a background of one ug of genomic human DNA. Assay specificity was evaluated using PBMC DNA from 41 anonymous US blood donors screened negative for HIV and HTLV.

Nested PCR was also performed at BSRI using double blinded genomic DNA specimens in order to independently test for XMRV *gag *sequences. The first round was performed as previously described to detect XMRV in PBMC DNA of CFS patients [11]. Briefly, 100 - 250 ng of genomic DNA was amplified using outer gag primers 419F (5' ATC AGT TAA CCT ACC CGA GTC GGA C 3') and 1154R (5' GCC GCC TCT TCT TCA TTG TTC TC 3') at a final concentration of 0.3 μM, HotStart-IT FideliTaq Master Mix (USB Corporation, Cleveland, OH) and 1 mM magnesium chloride. PCR was performed using an initial denaturation step at 94°C for 4 minutes followed by 45 cycles of 94°C for 30 seconds, 57°C for 30 seconds and 72°C for 1 minute and a final extension step at 72°C for 2 minutes. Nested PCR was conducted using 1 μl of the first round DNA in the second round reaction. Nested primers 488F (5' GGG GAC GAG AGA CAG AGA CA 3') and 1107R (5' CAG AGG AGG AAG GTT GTG CT 3') were used at a final concentration of 0.3 μM and amplification was performed using HotStart-IT FideliTaq. PCR was performed using an initial denaturation step at 95°C for 90 seconds followed by 40 cycles of 95°C for 20 seconds, 58°C for 30 seconds and 72°C for 40 seconds and a final extension step at 72°C for 2 minutes. PCR contamination occurring during nested PCR was evaluated by including at least one third as many water controls as test samples in each PCR experiment and were always negative.

Using serial dilutions of a cloned fragment of XMRV *gag *as a positive control, the nested PCR assay could reliably detect at least 3 copies of DNA per reaction, even when spiked into genomic DNA prepared either from 293FT cells or donor PBMCs previously validated to be negative for XMRV. Controls of GAPDH (forward - 5' CAT GTT CCA ATA TGA TTC AC 3'; reverse - 5' CCT GGA AGA TGG TGA TG 3'; 75 ng genomic DNA, 3 minutes at 95°C followed by 45 cycles of 95°C for 20 seconds, 55°C for 45 seconds and 72°C for 30 seconds, followed by 1 cycle of 72°C for 2 minutes) were performed to ensure similar levels of genomic DNA input in each PCR reaction.

## Competing interests

The authors declare that they have no competing interests.

## Authors' contributions

WMS, WCR, RMH and WH conceived and designed the study. WCR and VRF provided specimens and data on study population. HJ, HZ, ST, AS, GS, NB, and OH performed specimen testing and data analysis with WMS and WH. WMS, WCR and WH wrote the manuscript. All authors read and approved the final manuscript.
